# Baker’s Yeast-Based Microbial Fuel Cell Mediated by 2-Methyl-1,4-Naphthoquinone

**DOI:** 10.3390/membranes11030182

**Published:** 2021-03-06

**Authors:** Juste Rozene, Inga Morkvenaite-Vilkonciene, Ingrida Bruzaite, Antanas Zinovicius, Arunas Ramanavicius

**Affiliations:** 1 Department of Mechatronics, Robotics, and Digital Manufacturing, Faculty of Mechanics, Vilnius Gediminas Technical University, 03224 Vilnius, Lithuania; juste.rozene@vilniustech.lt (J.R.); antanas.zinovicius@vilniustech.lt (A.Z.); 2 Laboratory of Electrochemical Energy Conversion, State Research Institute Centre for Physical Sciences and Technology, 10257 Vilnius, Lithuania; 3 Department of Chemistry and Bioengineering, Faculty of Fundamental Sciences, Vilnius Gediminas Technical University, 10223 Vilnius, Lithuania; ingrida.bruzaite@vilniustech.lt; 4 Department of Physical Chemistry, Faculty of Chemistry and Geosciences, Vilnius University, 03225 Vilnius, Lithuania; 5 Laboratory of Nanotechnology, State Research Institute Centre for Physical Sciences and Technology, 02300 Vilnius, Lithuania

**Keywords:** baker’s yeast cells, *Saccharomyces cerevisiae*, microbial fuel cell, 2-methyl-1,4-naphthoquinone, biofuel cell, graphite rod electrode

## Abstract

Microbial fuel cell (MFC) efficiency depends on charge transfer capability from microbe to anode, and the application of suitable redox mediators is important in this area. In this study, yeast viability experiments were performed to determine the 2-methyl-1,4-naphthoquinone (menadione (MD)) influence on different yeast cell species (baker’s yeast and *Saccharomyces cerevisiae* yeast cells). In addition, electrochemical measurements to investigate MFC performance and efficiency were carried out. This research revealed that baker’s yeast cells were more resistant to dissolved MD, but the current density decreased when yeast solution concentration was incrementally increased in the same cell. The maximal calculated power of a designed baker’s yeast-based MFC cell anode was 0.408 mW/m^2^ and this power output was registered at 24 mV. Simultaneously, the cell generated a 62-mV open circuit potential in the presence of 23 mM potassium ferricyanide and the absence of glucose and immobilized MD. The results only confirm that MD has strong potential to be applied to microbial fuel cells and that a two-redox-mediator-based system is suitable for application in microbial fuel cells.

## 1. Introduction

A microbial fuel cell (MFC) is a bio-electrochemical appliance with microorganisms, which converts chemical energy into electric energy, mostly acting at the anode compartments [1,2]. The advantage of MFCs over enzymatic biofuel cells are that microbes are less susceptible to poisoning and loss of activity under normal operating conditions [3]. In MFCs, mixed cultures of microorganisms can be used, such as *R. ferrireducens*, *E. coli*, *Shewanella oneidensis*, or even a mixed community [4]. Baker’s yeast and *Saccharomyces cerevisiae* are used rarely but successfully [5,6]. MFCs are environmentally friendly and suitable for compensating the energy used to treat various industrial wastewater, and they do not require strict conditions [7,8]. MFCs’ output is restricted, while high internal resistance considerably decreases the energy produced by MFCs [9]. The main factor determining the element’s efficiency is the charge transfer from the microorganisms to the electrode [8]. This issue can be solved by adding suitable mediators and optimizing cell design and electrode. To be used in MFCs, a mediator with appropriate properties must be used. It must be electrochemically active, soluble, chemically stable, non-toxic to microorganisms, easily permeable to the cell membrane, and it must have a redox potential adequate for electron transfer with a rapid oxidation process at the electrode surface [8,10]. Some of the best known artificial mediators are thionine, methylene blue, 2-hydroxyl,4-naphthoquinone, thionine, meldola’s blue, and neutral red [8]. Conductive polymers can be chosen as the redox mediators [11]. In this case, the anode can be modified by conductive polymers, or living cells can perform the polymerization process. Anodes can be modified by nanomaterials or by both nanomaterials and polymers [12]. Carbon nanotubes (CN) can improve the charge transfer and the working area of the electrode. The composite of CN and polyaniline nanostructure might be used as the material for the anode electrode to achieve the best operating parameters to maintain the potential value [5]. The anode can be modified by lipophilic redox mediators to avoid organic solvents in solution and to ensure a redox mediator concentration that is as low as possible. In this case, it becomes possible to use more than just non-toxic redox mediators. In our earlier research, we showed that 9,10-phenantrenequinone [13,14] and 2-methyl-1,4-naphthoquinone (vitamin K3, menadione) [15,16] at low concentrations can be used as redox mediators. Menadione is reduced intracellularly and diffused back through the membrane into the solution [17,18]. The second redox mediator, such as ferricyanide, should be used to ensure effective electron transfer to the electrode [13]. This is called a two-mediator-based system: one mediator is lipophilic, which interacts with the intracellular redox centers; another mediator is hydrophilic, which takes an electron from the lipophilic mediator and passes it to the electrode [19]. Some of the quinones can be used as a lipophilic mediator and were associated with bone marrow toxicity; additionally, quinones formed by oxidation of polycyclic aromatic hydrocarbons may form adducts with DNA and RNA, and quinone compounds have been used as anti-cancer drugs [20,21,22,23]. Quinones can form reactive oxygen species (ROS), such as superoxide (O_2_^−^) or hydrogen peroxide (H_2_O_2_), which can be deleterious [20,22,24]. The effect of quinones on living cells is still unclear and depends on the conditions of cellular exposure.

This study aimed to evaluate the effects of 2-methyl-1,4-naphthoquinone exposure on the viability of the yeast *Saccharomyces cerevisiae* and the possibility to use this compound as the redox mediator in the MFC.

## 2. Materials and Methods

### 2.1. Materials

Commercially available baker’s yeast was purchased from a food supplier, Dr. Oetker Lietuva (Vilnius, Lithuania), and the wild type (21PMR) *Saccharomyces cerevisiae* yeast (MAT leu2 ura3-52) strain was obtained from Dr. E. Serviene (Nature Research Centre, Lithuania). The 0.5 M phosphate–acetate buffer solution (pH 6.77) was prepared by dissolving 0.05 M CH_3_COONa, 0.05 M NaH_2_PO_4_, 0.05 M Na_2_HPO_4_, and 0.1 M KCl in distilled water. All chemicals were purchased from Sigma-Aldrich (Steinheim, Germany). Glucose (≥98%) and potassium ferricyanide (≥99.0%) were prepared in the phosphate–acetate buffer solution, and both were purchased from Riedel (Vilnius, Lithuania). Glucose solution was mutarotated overnight before the investigation. 2-methyl-1,4-naphthoquinone (vitamin K_3_, menadione (MD)) was dissolved in distilled water. MD was purchased from Sigma-Aldrich (Steinheim, Germany). A graphite electrode for the anode and a Whatman^®^ nuclepore membrane for the anode covering used in the electrochemical experiments were purchased from Sigma-Aldrich (Steinheim, Germany).

### 2.2. The Preparation of Yeast Samples

*Saccharomyces cerevisiae* 21PMR (MAT leu2 ura3-52) yeast strain and baker’s yeast cells were grown in a liquid yeast extract peptone glucose (YPG) medium (1% (*w*/*v*) yeast extract, 2% (*w*/*v*) peptone, and 3% (*w*/*v*) glucose) on a rotary shaker at 120 rpm at 20 °C and 30 °C for 16 h. The culture of yeast cells was incubated on a Heidolph Unimax 1011 shaker with an incubator (Schwabach, Germany). For electrochemical and MFC measurements, baker’s yeast was prepared in this way: 500 mg of baker’s yeast was added to the 1 g suspension of YPD-broth mixed with 20 mL distilled water (concentration = 50 g/L). A further culture was grown on a rotary shaker at 200 rpm and 30 °C for 20 ÷ 24 h.

### 2.3. Graphite Electrode Preparation

A smaller piece of the 150 mm graphite electrode rod (ø3 mm, low density, 99,995% trace metal basis) was cut and sanded with paper with three different grinding bead sizes and washed with distilled water and 97% ethanol. A 2.5 µL drop of MD (3.75 mM) was added to the electrode. The 2.5 µL drop of the prepared yeast (0.125 g/mL) was immobilized on the dried electrode with or without MD.

### 2.4. The Preparation of Cells for Viability Assays

For the agar well diffusion method, the cultures of (i) *Saccharomyces cerevisiae* yeast and (ii) baker’s yeast was inoculated in the flask overnight (~16 h). The initial optical density (OD) of the culture at a 600 nm wavelength was 1.2 ± 0.01, which corresponds to 3.6 × 10^7^ of colony-forming units (CFU) per mL. Then, 0.1 mL of the yeast cell suspension was transferred to a 9 cm Petri dish, after that 10 mL of YPG agar medium was added to the same plate and mixed smoothly. The holes (~8 mm in diameter) were made on the cooled and frozen YPG agar medium. Then, 0.025 mL of 3.75 mM concentration 2-methyl-1.4-naphthoquinone solution was introduced in the well. Ethanol was used as a control solution. Petri dishes were incubated in a laminar airflow box (LaboGene ScanLaf Mars) (Lillerød, Denmark) at 20 °C and 30 °C for 24 h.

Regarding the determination of total cell number by culture optical density, for this experimental investigation, the *Saccharomyces cerevisiae* yeast cells and baker’s yeast cells were inoculated in the presence of 3.75 mM concentration 2-methyl-1.4-naphthoquinone solution. Yeast cells unaffected with MD were served as the control. 1 mL of overnight yeast cells cultures were transferred to 9 mL of fresh liquid YPG medium (optical density at 600 nm was ~0.1) and cultivated for 4 h at 30 °C and 5 h at 20 °C (OD at 600 nm was 0.51 ÷ 0.55). After that, 100 µL of MD solution was added to the prepared yeast cell suspensions and the suspensions were incubated at 20 °C and 30 °C for 26 h. The OD cultures of the yeast cells were determined within 26 h at 2-h intervals between measurements. Yeast cell numbers were monitored on liquid YPG media by measuring the light absorbance by the YPG medium sample with the cells using a Genesys 10S UV-VIS spectrophotometer from Thermo Fisher Scientific (Mettler Toledo, Singapore).

### 2.5. Electrochemical Measurements

Electrochemical measurements were performed using an Autolab PGSTAT 30 Potentiostat/Galvanostat (Utrecht, the Netherlands) and NOVA software. All experiments were carried out at ambient temperature (at 20 °C) while stirring in-between the phosphate–acetate buffer solution measurements, pH = 6.77, under aerobic conditions. The cycles were measured five times, and the last one was plotted in the results section. If the concentration of additional material was changed, it was done sequentially by adding it to the same measurement cell. All the measurements were repeated 5 times, and the values were derived.

Measurements were performed in a three-electrode electrochemical cell, where the graphite electrode was connected as a working electrode, the platinum electrode as a counter electrode, and the Ag/AgCl/KCl (3M) electrode as a reference electrode. All the components (borosilicate glass titration vessel with plastic mounting ring and lid, platinum, and 12.5 cm length Ag/AgCl/KCl (3M) electrodes) were purchased from Metrohm AG (Herisau, Switzerland). Cyclic voltammograms were recorded at a scan rate of 0.1 V/s in the range from −0.6 V to 0.8 V.

A two-electrode-based electrochemical cell was used for the study of the MFC performance. An MD/yeast-modified graphite electrode was used as the anode, and a bare graphite electrode was used as the cathode. A track-etched polycarbonate membrane with a 3 µm pore size was used for the covering of the anode surface. During voltage measurements of the MFC, the external resistances (of 0.01 kΩ, 0.1 kΩ, 0.42 kΩ, 0.9 kΩ, 5 kΩ, 12 kΩ, 50 kΩ, 100 kΩ, 470 kΩ, 1100 kΩ, 2100 kΩ, and 2600 kΩ) were plugged into an external electrical circuit to imitate the load and to assess the power density of the designed MFC anode.

### 2.6. Calculations

Electrochemical measurements were evaluated using Hill’s function:

(1) $$J=\frac{C^{n}}{k^{n}+C^{n}}$$

where: J is current density, C is the concentration of selected substrate (glucose, MD, or potassium ferricyanide), k is the half-maximal concentration constant, and n is the Hill coefficient.

The modified Hill’s function was used for the evaluation of time-dependent experiments:

(2) $$J=\frac{t^{n}}{k^{n}+t^{n}}$$

where: J is current density, t is the reaction time, k is the half-maximal concentration constant, and n is the Hill coefficient.

## 3. Results and Discussion

### 3.1. The Assessment of Yeast Cells’ Viability

**Figure 1:**
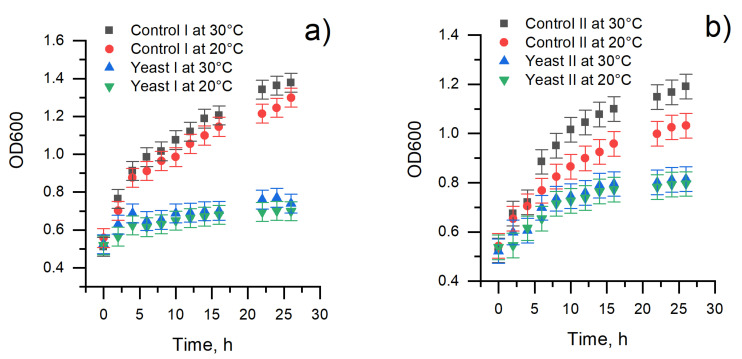
Variation of optical density (OD) during the determination of growth curves of yeast. (**a**) A comparison of growth curves: (i) yeast I—*Saccharomyces cerevisiae* with 3.75 mM menadione (MD); control I—*Saccharomyces cerevisiae* in the liquid YPG agar medium; (**b**)—a comparison of growth curves: (ii) yeast II—baker’s yeast with 3.75 mM MD; control II—baker’s yeast in the liquid YPG agar medium.

In viability studies, it is crucial to determine the critical time after which a cell number change during cultivation is observed. The growth of yeast cells was assessed by determining the optical density of the cells’ suspension at 600 nm. Measurements of the optical density of baker’s yeast and *Saccharomyces cerevisiae* yeast cells grown in the liquid YPG medium with or without MD solution are shown in Figure 1.

We cultured different yeast cell species and determined that the optical density of the cells’ suspension increased with increasing temperature and by prolonging the time of cultivation. The optimal temperature for *Saccharomyces cerevisiae* 21PMR (MAT leu2 ura3-52) yeast strain grows at 30 °C temperature (Figure 1a, control I). However, baker’s yeast (Figure 1b, control II) multiplied more slowly; it depends on the species of yeast cells. When yeast cells were cultivated with 3.75 mM of MD, optical density changed differently for both species. OD slightly decreased after 4 h and 26 h cultivation of *Saccharomyces cerevisiae* yeast strain (Figure 1a, yeast I), but, baker’s yeast cells still increased after 4 h and 26 h cultivation (Figure 1b, yeast II). The viability decreased for both species of yeast cells. This result may indicate cell death due to adverse growing conditions, which was due to the toxic effect of 2-methyl-1,4-naphthoquinone (vitamin K3) [25,26]. The growth curves of both yeast aliquots showed that baker’s yeasts are more resistant towards dissolved MD than *Saccharomyces cerevisiae* yeast cells.

**Figure 2:**
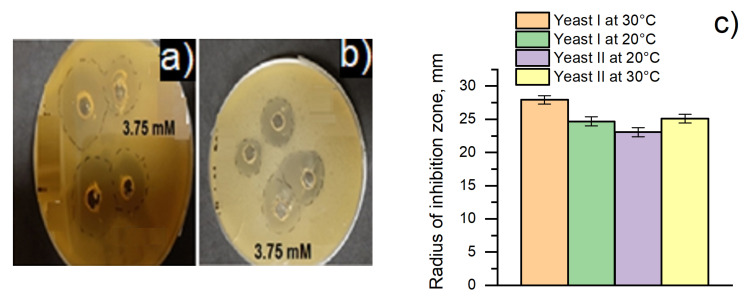
Antimicrobial activity of MD solution evaluated by the agar well diffusion method against yeast cells: (**a**)—yeast I ((i) *Saccharomyces cerevisiae*) at 30 °C); (**b**)—yeast II ((ii) baker’s yeast) at 30 °C; (**c**)—the histogram presents the radius of the inhibition zone at 3.75 mM concentration of MD in the solution.

The influence of 3.75 mM MD solution on yeasts’ viability was investigated against (i) *Saccharomyces cerevisiae* yeast and (ii) baker’s yeast cells. Figure 2 represents the radius of the inhibition zone of both yeast cells cultivated at 20 °C and 30 °C (Figure 2a,b). In the solution containing 3.75 mM of MD, inhibition zones were recorded, which indicated antimicrobial activity dependent on the species of yeast cell and the temperature of cultivation. The results of the antimicrobial activity of the MD solution are shown in Figure 2c. The radius of zones of inhibition for *Saccharomyces cerevisiae* yeast cells and baker’s yeast cells was determined as 27.9 ± 0.6 mm (at 30 °C) and 24.7 ± 0.7 mm (at 20 °C) and 25.0 ± 0.6 mm (at 30 °C) and 23.1 ± 0.7 mm (at 20 °C), respectively. Considering that the radius of the zone of inhibition was the measure of antimicrobial activity, baker’s yeast cells were more resistant to dissolved 2-methyl-1,4-naphthoquinone. For this reason, baker’s yeast cells were selected for further development of MFCs.

### 3.2. The Evaluation of the MD-Modified Graphite Electrode at Different Yeast Concentrations

**Figure 3:**
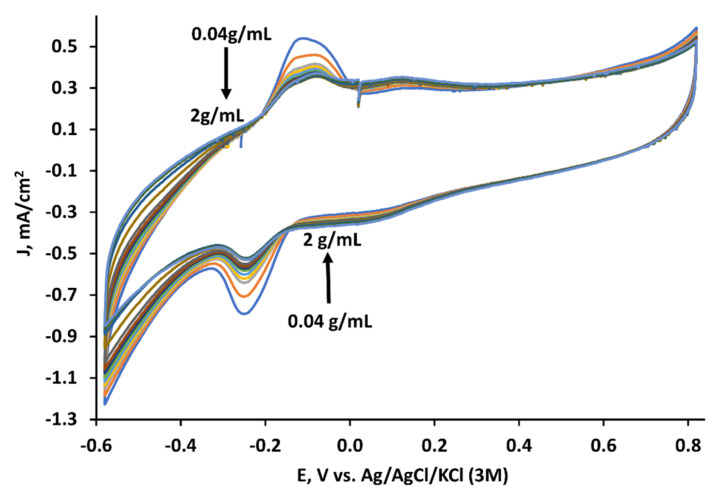
Cyclic voltammograms recorded with the MD-modified anode at different yeast concentrations in the phosphate–acetate buffer solution. The scan rate was 0.1 V/s.

Cyclic voltammograms were recorded while yeast solution concentration was being incrementally increased in the same cell. In this experiment, only MD was immobilized on the anode. The concentration of yeast solution was sequentially increased in the same measurement cell with the phosphate–acetate buffer solution to 2 g/mL (Figure 3). The current density decreased from 0.55 to 0.4 mA/cm^2^ at −0.15 V and from −0.8 to −0.52 mA/cm^2^ at −0.25 V potentials, while the yeast concentration increased from 0.04 to 2 g/mL.

### 3.3. The Evaluation of the Yeast-Modified Graphite Electrode at Different MD Concentrations

**Figure 4:**
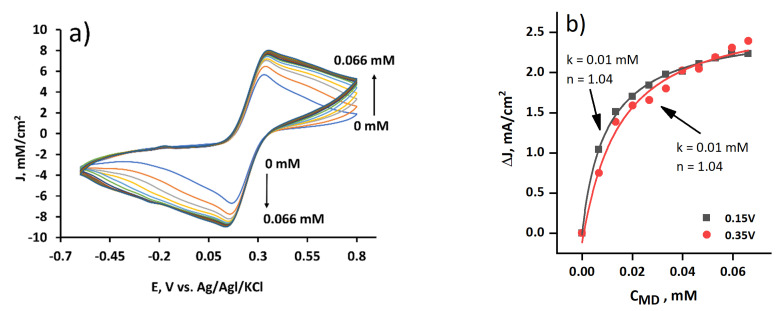
(**a**) Cyclic voltammograms registered by the yeast-modified graphite electrode at different MD concentrations. Measurements were performed in the phosphate–acetate buffer solution with the 37 mM potassium ferricyanide and 31 mM glucose. A scan rate of 0.1 V/s and a potential step of 0.01 V was applied; (**b**) reduction peak potentials at 0.15 V and 0.35 V vs. used MD concentration recorded using the yeast-modified graphite electrode (results were fitted using Hill’s function (Equation (1)). Measurements were performed in a three-electrode-based electrochemical cell.

The cyclic voltammograms of the yeast-modified graphite electrode were recorded with the potassium ferricyanide and the glucose in the phosphate–acetate buffer solution with different menadione concentrations (Figure 4a). The current density increased evenly in the range from 0.0 mM to 0.066 mM.

The peaks of current from the voltammogram were plotted as MD concentration dependence and evaluated by fitting Hill’s equation (Equation (1)) (Figure 4b). At the 0.15 V potential, the Hill coefficient *n* was less than one (*n* = 0.94). This meant a negative cooperativity for substrate binding, i.e., if the concentration of MD increases, then the affinity of other MD molecules decreases. At the 0.35 V potential, *n* was higher than one (*n* = 1.04), and this indicated a positive cooperativity, i.e., if MD concentration increases, then the efficiency of charge transfer by MD molecules also increases. The half-maximal concentration constant *k* was low (*k* = 0.01 mM) at both potentials, which meant that the reaction rate was speedy.

### 3.4. The Evaluation of the Yeast and MD-Modified Graphite Electrode at Different MD Concentrations

**Figure 5:**
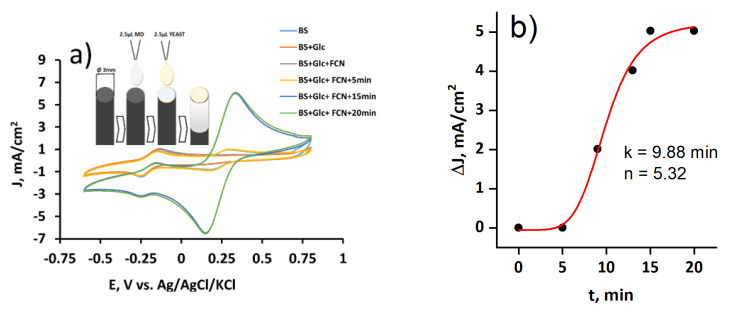
(**a**) Cyclic voltammograms of graphite electrode with immobilized MD and yeast cells registered in the phosphate–acetate buffer solution (BS) with the presence and absence of glucose (Glc) and potassium ferricyanide (FCN); (**b**) reduction peak current density at 0.4 V vs. used MD concentration recorded using an immobilized MD- and yeast-modified graphite electrode (results were fitted using Hill’s function (Equation (2)). Measurements were performed in a three-electrode-based electrochemical cell in the phosphate–acetate buffer solution with 23 mM glucose and 23 mM potassium ferricyanide.

The graphite electrode modified with MD and yeasts was used as a working electrode in the electrochemical cell, and cyclic voltammograms were recorded with and without glucose (Figure 5a). Cyclic voltammograms, observed in the phosphate–acetate buffer solution with and without glucose were similar. The MD oxidation–reduction peaks were obtained since MD was immobilized on the electrode (see Figure 5a, upper-left corner). We used the second redox mediator, potassium ferricyanide, to achieve a higher electrochemical signal and to transfer charge from yeast to the electrode. It lasted 15 min to achieve steady-state conditions for yeast. Our previous research used another quinone, 9,10-phenantrenequinone, as a lipophilic redox mediator, and the steady-state condition was observed after 20 min [13].

Peaks from time-dependent cyclic voltammograms (not shown) were plotted as time dependence and evaluated by fitting Hill’s function (Equation (2)) (Figure 5b). The Hill coefficient (*n*) was higher than one (*n* = 5.32), which meant positive cooperativity. The half-maximal concentration constant *k* was high (*k* = 9.88), so the reactions were slow. Comparing to the only yeast-modified electrode, the Hill coefficient range became five times higher (from *n* = 0.94 and 1.04 measured with the yeast-modified electrode (Figure 4b) to *n* = 5.32 obtained with the yeast- and MD-modified electrode). Additionally, compared to the only yeast-modified electrode, the range of the half-maximal concentration constant *k* became 1000 times higher (from *k* = 0.01 mM measured with the yeast-modified electrode (Figure 4b) to *k* = 9.88 obtained with the yeast- and MD-modified electrode).

Comparing the data from all experiments, it could be concluded that the MFC will generate the highest current at 0.15 V and 0.35 V (the oxidation and reduction potentials of potassium ferricyanide, respectively). The most significant change in current was seen when the second redox mediator was added to the solution. The toxic effects of MD for yeast were not obtained. Therefore, both of them are suitable for the design of biofuel cells.

**Figure 6:**
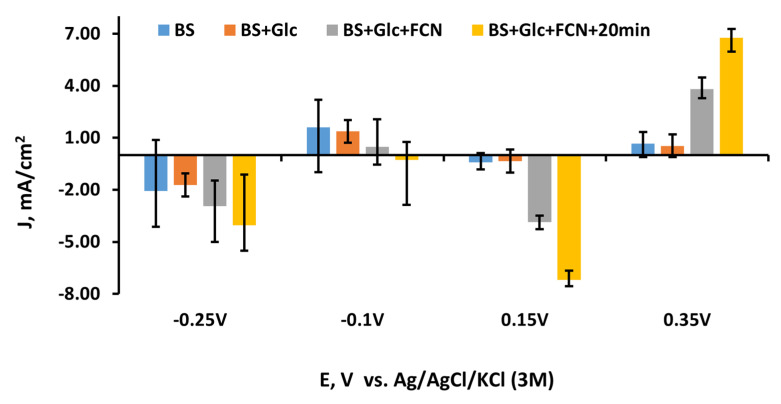
Data from replicated experiments with the MD and yeast-modified electrode.

The diagram of experiments using the MD-modified graphite electrode shows the change of current density at the different stages of the process: before and after adding new material to the solution and after the stabilization in current density was stopped (Figure 6). The current density after adding glucose was lower at −0.1 V potentials but similar at 0.15 V and 0.35 V potentials. A rather significant difference was observed between negative and positive potentials. Potassium ferricyanide provided almost the same current density value at all potentials, except −0.1 V. At potentials −0.25, 0.15, and 0.35 V positive and negative values of current densities increased over time, but at the −0.1 V potential, the current density value decreased when potassium ferricyanide was added. This result allowed observation of the interaction of these two mediators.

### 3.5. The Performance of the Microbial Fuel Cell

**Figure 7:**
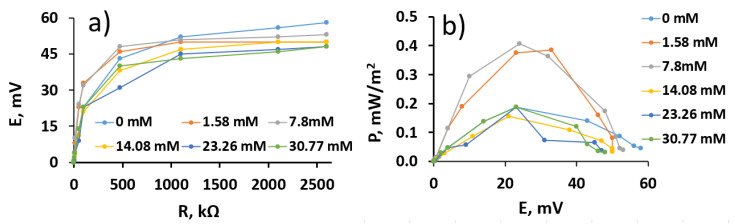
(**a**) Potential dependence on applied load; (**b**) calculated power density dependence on the generated potential in a single-compartment-based Microbial fuel cell (MFC), which consisted of an MD- and yeast-modified anode and a bare graphite cathode immersed in the phosphate–acetate buffer solution containing 23 mM of potassium ferricyanide and variable concentrations of glucose: 0 mM, 1.58 mM, 7.8 mM, 14.08 mM, 23.26 mM, and 30.77 mM.

To assess the MFC performance, a two-electrode-based electrochemical cell was used with the MD- and yeast-modified graphite electrode used as the anode and a bare graphite electrode as the cathode. It was determined that MFC, in which the anode was modified by MD and yeast, generated 58 mV potential in the absence of glucose at an external load of 2.6 MΩ and resulted in 0.0046 mW/m^2^ of power (Figure 7a). The maximal open circuit potential was 62 mV at 23 mM of potassium ferricyanide and the absence of glucose. The maximal calculated power was 0.408 mW/m^2^ at 24 mV (Figure 7b).

In our previous research, we evaluated the performance of MFC based on baker’s yeast and a 9,10-phenantrenequinone- (PQ) modified anode [6]. This MFC generated the 22.2 mW/m^2^ of power at 56 mV in the presence of 30 mM glucose. The maximal open circuit potential was 178 mV at 7.8 mM of glucose and 23 mM of potassium ferricyanide and PQ. Therefore, it can be concluded that menadione as well as PQ can be used in MFCs as redox mediators. However, PQ has a better ability to cross the yeast cell membrane and, thus, a PQ-based MFC results in higher power than an MD-based MFC.

## 4. Conclusions

An MFC anode based on MD and yeast cells (baker’s yeast and a pure *Saccharomyces cerevisiae* strain) was designed and evaluated. This research showed that baker’s yeast compared to *Saccharomyces cerevisiae* cells are more resistant towards MD exposure at a 30 °C temperature of cultivation. A two-redox-mediator-based system is suitable for charge transfer from yeast towards the electrode. The maximal calculated power of the designed baker’s yeast-based MFC anode was 0.408 mW/m^2^, and this power output was registered at 24 mV. Simultaneously, the cell generated a 62-mV open circuit potential in the presence of 23 mM potassium ferricyanide and the absence of glucose and immobilized MD. Therefore, it can be concluded that MD has strong potential to be applied to microbial fuel cells. It should be noted that we used bare graphite as a cathode, which significantly reduced the power of the designed MFC. Our future research will be related to the development of a cathode more suitable for this kind of MFC. Present research shows that a two-redox-mediator-based system is well suited for application in *Saccharomyces cerevisiae*-based MFCs.

## Data Availability

Data available upon request.
